# Complication Management for Transcatheter Tricuspid Annuloplasty Dislocation

**DOI:** 10.1016/j.jaccas.2025.105422

**Published:** 2025-10-25

**Authors:** Vasileios Exarchos, Andi Rroku, Mario Kasner, Ulf Landmesser, Gudrun Feuchtner, Anna Sannino, Markus Reinthaler, Fabian Barbieri

**Affiliations:** a Department of Cardiology, Angiology and Intensive Care Medicine, Deutsches Herzzentrum der Charité, Campus Benjamin Franklin, Berlin, Germany; b Department of Cardiology, Charité–Universitätsmedizin Berlin, Corporate Member of Freie Universität Berlin and Humboldt-Universität zu Berlin, Berlin, Germany; c Berlin Institute of Health at Charité–Universitätsmedizin Berlin, BIH Biomedical Innovation Academy, Berlin, Germany; d DZHK (German Centre for Cardiovascular Research), Partner site Berlin, Berlin, Germany; e Department of Radiology, Medizinische Universität Innsbruck, Innsbruck, Austria; f Institute of Active Polymers and Berlin-Brandenburg Center for Regenerative Therapies, Helmholtz-Zentrum Hereon, Teltow, Germany

**Keywords:** chronic heart failure, computed tomography, echocardiography, hybrid imaging, imaging, insufficiency, three-dimensional imaging, tricuspid valve, valve repair, valve replacement

## Abstract

Severe secondary tricuspid regurgitation (TR) is increasingly recognized as a major contributor to heart failure symptoms and mortality, particularly in elderly patients with complex comorbidities. Transcatheter tricuspid valve interventions have emerged as promising alternatives for these challenging cases. We report the cases of 2 patients with severe dyspnea and leg edema who were diagnosed with torrential TR. Both patients initially underwent transcatheter annuloplasty using the Cardioband system (Edwards Lifesciences), which led to significant improvements in TR severity and clinical symptoms. However, both experienced device dislocation and recurrent torrential TR within months. Subsequent complication management included orthotopic and heterotopic transcatheter tricuspid valve replacement, resulting in marked clinical improvement. This report underscores the potential and challenges of transcatheter therapies for severe functional TR, highlighting the importance of careful patient selection, close monitoring, and adaptive treatment strategies to optimize outcomes.

## Graphical Abstract

**Figure:**
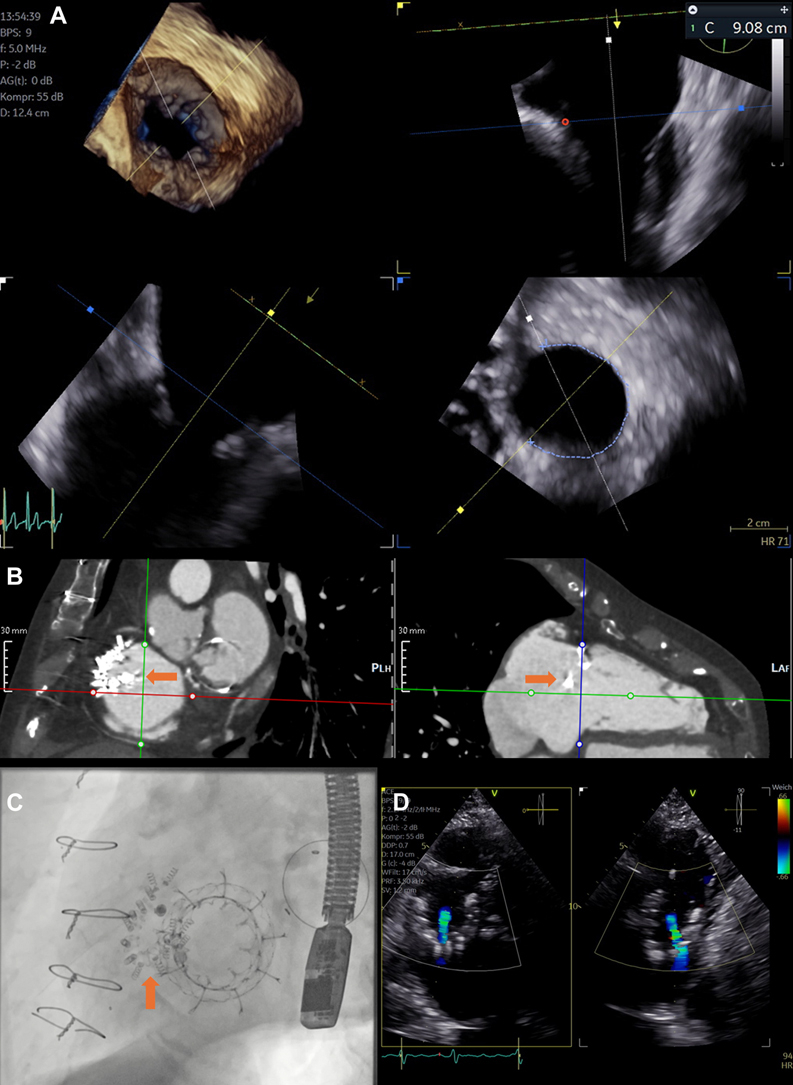

## Patient 1

An 84-year-old woman with a history of permanent atrial fibrillation, surgical mitral valve replacement, and heart failure with preserved ejection fraction presented to our clinic with recurrent episodes of cardiac decompensation. Symptoms included exertional dyspnea (NYHA functional class III-IV) and bilateral lower extremity edema.

Echocardiographic evaluation revealed a newly reduced left ventricular ejection fraction (LVEF) of 40%, borderline preserved right ventricular function (tricuspid annular plane systolic excursion [TAPSE]: 17 mm), significant biatrial dilatation, and normal function of the biological mitral valve prosthesis. Further evaluation showed torrential tricuspid regurgitation (TR) of a predominantly ventricular functional type (vena contracta: 22 mm, effective regurgitation orifice area [EROA]: 1.2 cm^2^, triangular continuous wave Doppler signal, systolic flow reversal into the hepatic veins) (Videos 1 and 2). Right heart catheterization revealed postcapillary pulmonary hypertension (mean pulmonary artery pressure: 22 mm Hg, mean wedge pressure: 16 mm Hg, pulmonary vascular resistance: 1.7 Wood units) and low cardiac output (3.5 L/min, cardiac index: 2.0 L/min/m^2^). Cardiac computed tomography angiography (CCTA) was conducted to assess the feasibility of different transcatheter tricuspid valve intervention (TTVI) devices.

**Figure 1:**
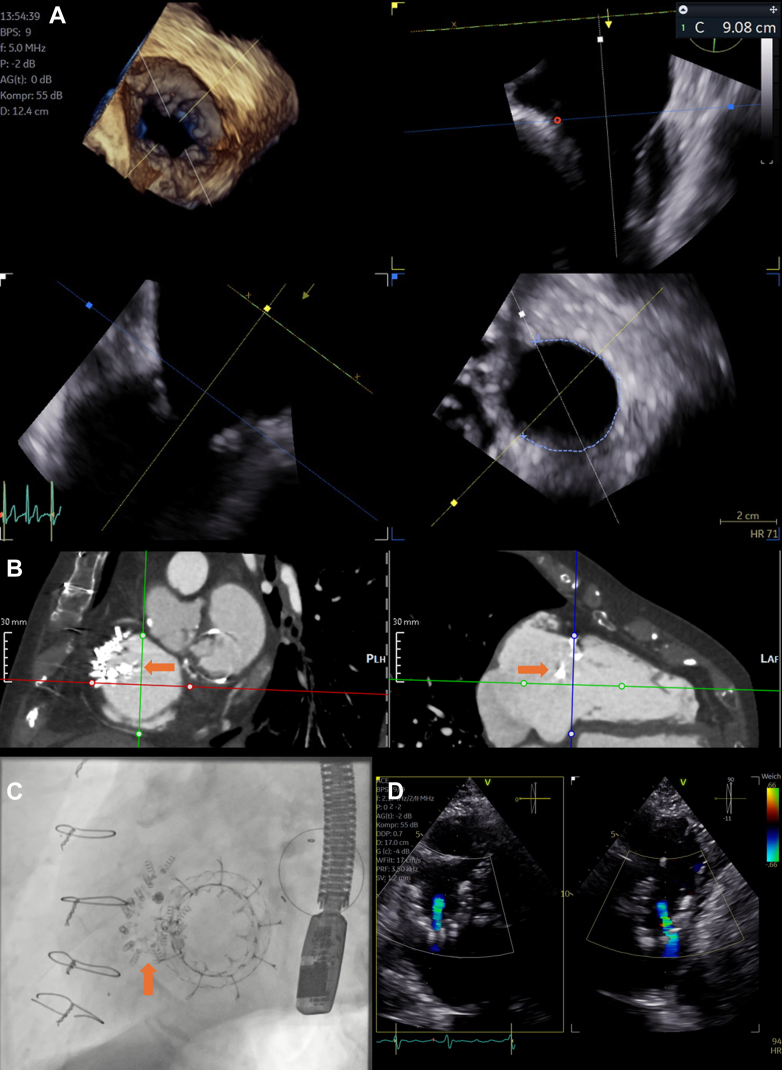
Pre-, Peri- and Postinterventional Imaging for Patient 1 (A) Three-dimensional transesophageal echocardiography demonstrates annuloplasty coverage measurement of the tricuspid annulus, starting anteriorly, as close to the aorta as possible, right to the opening of the coronary sinus. (B) Cardiac computed tomography angiography short-axis and long-axis views demonstrate dislocation of the annuloplasty device (orange arrows). (C) Fluoroscopic view at the end of the procedure with the dislocated annuloplasty device (orange arrow) and orthotopic transcatheter tricuspid valve replacement. (D) Transthoracic echocardiography reveals final result with mild to moderate prosthetic valve regurgitation.

During discussion within the heart team, the patient was deemed inoperable owing to her age and comorbidities. Interventional therapy with the Cardioband system (Edwards Lifesciences) was chosen because of her favorable anatomy and successful screening. The procedure was conducted under general anesthesia with transesophageal echocardiographic guidance. A D-sized band was implanted by positioning 14 anchors (Figure 1A) around the tricuspid annulus and confirming adequate tissue insertion by pull tests, which were verified directly or indirectly by fluoroscopy and echocardiography (Video 3, Video 4, Video 5). Anchor 11 was skipped given its proximity to the right coronary artery. The device was cinched to the maximum of 5.0 as per the instructions for use, which resulted in moderate residual TR (Video 6, Video 7, Video 8). Discharge echocardiography confirmed the reduction in TR severity (vena contracta: 6 mm).

Six months later, the patient presented with worsening symptoms (NYHA functional class III-IV). Echocardiography showed significant right ventricular dysfunction (TAPSE: 10 mm) and recurrent torrential TR due to posterior and lateral anchor tear-out, leading to Cardioband detachment (Figure 1B) and loss of device function (Videos 9 and 10). Despite intensified intravenous diuretic therapy, there was minimal improvement on echocardiographic findings. Shortly before discharge, severe to massive TR persisted. After additional multidisciplinary heart team discussion and repeat CCTA, the patient was found to be eligible for orthotopic transcatheter tricuspid valve replacement (TTVR). Accordingly, a 44-mm Evoque valve (Edwards Lifesciences) was implanted, without any notable complications (Figure 1C, Video 11, Video 12, Video 13, Video 14, Video 15). Postprocedural echocardiography demonstrated normal valve function without stenosis or paravalvular leakage, but mild to moderate valvular regurgitation was noted (Figure 1D, Video 16).

The patient returned for routine follow-up evaluation after valve implantation. Clinically, the lower extremity edema had resolved entirely, although the patient reported persistent exertional dyspnea (NYHA functional class III). Echocardiographic assessment revealed satisfactory prosthetic tricuspid valve function, confirming previously noted mild to moderate valvular regurgitation. However, significant left ventricular dysfunction was noted (LVEF approximately 30%). Electrocardiography showed atrial fibrillation with tachyarrhythmia absoluta. The decision was made to intensify heart rate control therapy with digitoxin. Given the decline in biventricular function, cautious intensification of heart failure therapy was conducted. Spironolactone and an angiotensin-converting enzyme inhibitor were added to the treatment regimen, with close monitoring of blood pressure owing to the patient's predisposition to hypotension. These adjustments resulted in adequate rate control and further improvement of her symptoms (NYHA functional class II) as confirmed by telephone consultation.

## Patient 2

An 87-year-old woman with a history of permanent atrial fibrillation, chronic kidney disease stage 4, and heart failure with preserved ejection fraction presented with dyspnea at exertion (NYHA functional class II) and recurrent episodes of bilateral lower extremity edema with concomitant stasis dermatitis, consistent with recurrent cardiac decompensation.

Transthoracic and transesophageal echocardiography revealed preserved LVEF (57%), mildly to moderately reduced right ventricular systolic function (TAPSE: 15 mm) and torrential mixed functional TR (vena contracta: 23 mm, EROA: 0.96 cm^2^) (Video 17). Further quantitative assessment revealed marked right ventricular dilation (basal right ventricular end-diastolic diameter: 51 mm) and substantial tricuspid annular dilation (49 mm) (Video 18). Right heart catheterization revealed combined precapillary and postcapillary pulmonary hypertension (mean pulmonary artery pressure: 36 mm Hg, mean wedge pressure: 25 mm Hg, pulmonary vascular resistance: 2.18 WU) with normal cardiac output (4.56 L/min, cardiac index: 2.48 L/min/m^2^). CCTA was conducted to assess the feasibility of TTVI devices.

**Figure 2:**
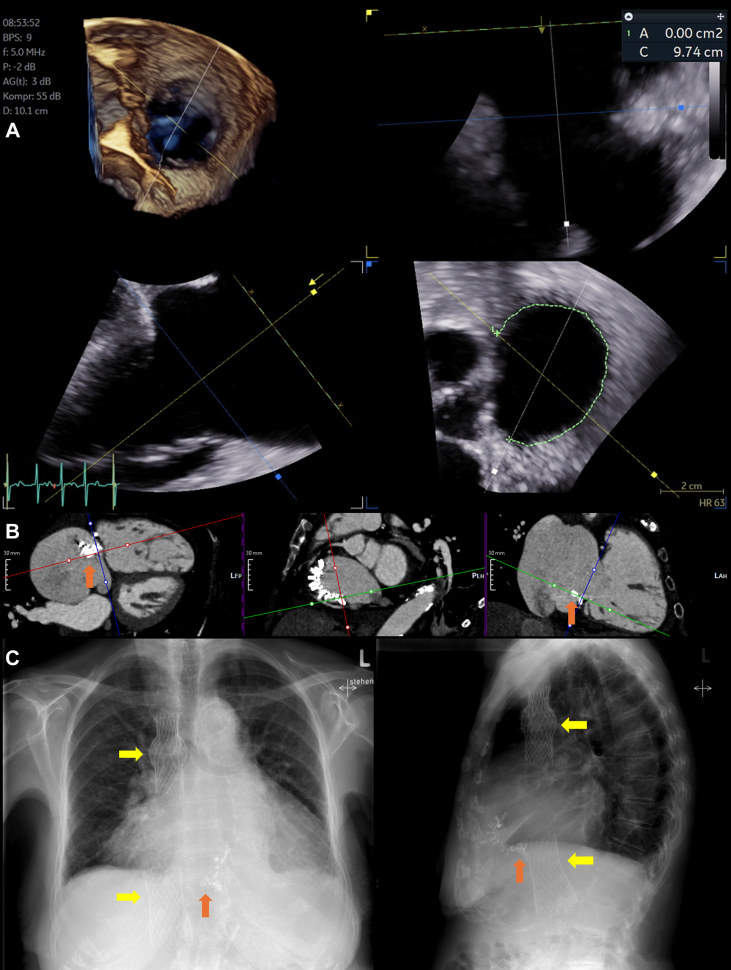
Pre-, Peri- and Postinterventional Imaging for Patient 2 (A) Three-dimensional transesophageal echocardiography demonstrates annuloplasty coverage measurement of the tricuspid annulus, starting anteriorly, as close to the aorta as possible, right to the opening of the coronary sinus. (B) Cardiac computed tomography angiography multiplanar reconstruction demonstrates Cardioband dislocation of the annuloplasty device (orange arrows). (C) Postprocedural chest radiography (anteroposterior and lateral views) show placement of the superior vena cava and inferior vena cava valves (yellow arrows) as well as the dislocated annuloplasty device (orange arrows).

After comprehensive discussion within our interdisciplinary heart team, the patient was deemed inoperable owing to her age, frailty, and comorbidities. As her anatomy was favorable and screening results positive, the she successfully underwent Cardioband implantation (E-sized band), and 15 anchors were placed in conventional manner (Figure 2A). Anchor 11 was skipped owing to right coronary artery proximity, and pull tests were performed to confirm adequate placement of every other anchor before release (Video 19, Video 20, Video 21). The device was then cinched to the maximum of 5.5 (Video 22, Video 23, Video 24). Echocardiographic evaluation demonstrated marked reduction in TR severity, which was classified as severe (Video 25).

Four months postprocedure, the patient presented with worsening dyspnea (NYHA functional class III-IV) and recurrent lower extremity edema. Echocardiographic assessment revealed a mildly reduced LVEF (45%). Additionally, distal anchor dislocation (Figure 2B) was observed, causing loss of annuloplasty effect and resulting in recurrent torrential TR. Despite escalation of intravenous diuretic therapy, there was limited improvement in the patient's clinical condition and echocardiographic parameters. Unfortunately, the possibility of diuretic escalation was also limited by the presence of worsening chronic renal failure. CCTA was repeated to evaluate alternative therapy strategies. After thorough evaluation and discussion in our interdisciplinary heart team conference, the patient underwent successful heterotopic TTVR using the transcatheter bicaval TricValve system (Products & Features), without any notable complications. According to CCTA measurements, a 25-mm valve was implanted into the superior vena cava, and a 31-mm valve was chosen for the inferior vena cava (Figure 2C, Video 26).

At follow-up, there was a marked clinical improvement, with significant regression of the lower extremity edema. Furthermore, the previous decline in renal function had also stabilized.

## Discussion

Severe TR is recognized as a critical contributor to heart failure symptoms, particularly among elderly patients with complex comorbid conditions. Functional TR accounts for over 90% of cases, primarily driven by either tricuspid annular dilation and/or right ventricular remodeling, rather than primary structural abnormalities of the valve leaflets.^1^ Surgical intervention is often contraindicated in these patients owing to prohibitive procedural risk, whereas conventional pharmacological therapy, including oral and intravenous diuretics, typically yields limited efficacy. TTVIs have emerged as promising alternatives, particularly in patients with symptomatic, refractory TR.^2^

One type of TTVI is the Cardioband transcatheter annuloplasty system, which offers a comprehensive solution by directly targeting annular dilation, yielding good procedural results in both atrial and ventricular functional TR.^3^^,^^4^ The device is thought to reduce annular dimensions, restore leaflet coaptation, and preserve native valve anatomy. Despite these advantages, patient selection remains a cornerstone of this therapy. Consecutive screening of patients by CCTA demonstrated that approximately 60% of patients are suitable for therapy, as excessive annular dilation, cardiac implantable electronic leads, right coronary artery proximity, and inferior vena cava insertion are the main reasons for screening failure.^5^ Moreover, significant functional challenges seem to persist in terms of tissue quality to securely place anchors around the annulus.

Both patients in this report experienced distal device dislocation during short-term follow-up, resulting in recurrent torrential TR and marked clinical decompensation. Interestingly however, the anchor dislocation did not result in any other complications such as annular perforation or pericardial effusion, but only in loss of therapeutic effect. To mitigate this complication, preventive measurements may be taken by trying to maintain postprocedural euvolemia and avoiding early diuretic reduction, which could contribute to annular strain. Once device dislocation is detected, other forms of TTVI were found in the form of TTVR to stabilize both patients. In the first case, we were able to demonstrate the utility of orthotopic TTVR despite the presence of the dislocated band, which was located directly in front of the valve. There was hardly any interference between the devices during the implantation, but we did encounter a minor form of impaired imaging quality due to shadowing. Preprocedural planning included strategies to possibly maneuver the dislocated annuloplasty device with a steerable sheath. Similar to findings described in randomized controlled trials, replacement of the valve led to marked improvement in symptoms, which was most pronounced in terms of edema regression.^6^

In the second case, heterotopic TTVR led to patient stabilization. Conversely to orthotopic TTVR, no immediate interference was anticipated in preprocedural planning. Hence, the procedure was conducted in a typical manner, without any specific considerations regarding the dislocated annuloplasty device. The strategy itself to consider caval valve implantation as a bailout option is already described in the literature. Previous case reports have demonstrated the feasibility in patients with failed tricuspid transcatheter edge-to-edge repair (T-TEER) due to single leaflet device attachment.^7^^,^^8^ Beyond the effect of heterotopic TTVR on symptoms, which was also described in the TRICUS EURO trial, we were also able to observe a stabilization in chronic renal failure progression.^9^ The hypothesis here is that the implantation of the inferior vena cava valve led to a reduction in abdominal congestion and renal tamponade, reducing the compression of the renal structures.^10^

Drawing a direct comparison between failed T-TEER and dislocated annuloplasty device, another distinctive feature of transcatheter annuloplasty devices becomes visible. Here, the integrity of the valve itself remains intact, as only the annulus is targeted, leaving the option for additional orthotopic TTVR or also T-TEER itself. This strategy is also used if marked TR or mixed etiologies (eg, annular dilatation and leaflet prolapse) is seen during preprocedural screening, and staged implantation, often referred to as “COMBO therapy,” is performed to achieve better and more durable results.^11^^,^^12^ In case of transcatheter annuloplasty dislocation, adjunctive therapies remain possible as shown, yet its part in the therapeutical effect is lost.

## Conclusions

This report underscores the importance of structured, intensive follow-up protocols to detect early device instability or recurrent TR. Proactive monitoring using multimodal imaging and clinical assessments enables timely reintervention, potentially preventing significant clinical deterioration. Furthermore, the progressive decline in biventricular function observed in our patients emphasizes the need for ongoing optimization of heart failure management. Careful titration of medical therapy may improve cardiac function while minimizing hemodynamic compromise in this vulnerable population.

## Ethics Statement

This report was approved by the ethics committee of Charité–Universitätsmedizin Berlin (EA4/013/21).

## Funding Support and Author Disclosures

Dr Barbieri has received grant support from Abbott Laboratories and Boston Scientific, consulting fees from Boston Scientific and Edwards Lifesciences, and speaking honoraria from Edwards Lifesciences. Dr Sannino has received research grants from Edwards Lifesciences and Venus Medtech. All other authors have reported that they have no relationships relevant to the contents of this paper to disclose.

**Take-Home Messages**

• Transcatheter annuloplasty therapy can effectively reduce secondary tricuspid regurgitation, but device dislocation with loss of therapeutic effect is a significant risk.
• Alternative therapies, such as orthotopic transcatheter tricuspid valve replacement and heterotopic caval valve implantation, can offer effective solutions in cases of device failure or complicated tricuspid regurgitation.
• Careful patient selection, continuous monitoring, and an adaptive treatment strategy are essential for optimizing outcomes in elderly patients with severe TR and heart failure, especially those with multiple comorbidities.

## Appendix

Video 1 Patient 1: Preprocedural Transesophageal Short-Axis Biplane View With Color Doppler

Video 2 Patient 1: Preprocedural Transesophageal Short-Axis Biplane Sweep

Video 3 Patient 1: Transgastric Long-Axis View of the Tricuspid Valve During With Adequate Anchor Positioning in Tissue (Anchor 12)

Video 4 Patient 1: Pull Test After Implantation of Anchor 13

Video 5 Patient 1: Pull Test After Implantation of Anchor 14

Video 6 Patient 1: Transesophageal Short-Axis Biplane View With Color Doppler After Transcatheter Annuloplasty Device Implantation

Video 7 Patient 1: Three-Dimensional En Face View of the Tricuspid Valve After Cinching

Video 8 Patient 1: Transgastric Short-Axis Biplane Sweep After Cinching

Video 9 Patient 1: Three-Dimensional En Face View of the Tricuspid Valve After Dislocation of the Annuloplasty Device

Video 10 Patient 1: Transesophageal Short-Axis Biplane View With Color Doppler After Dislocation of the Annuloplasty Device

Video 11 Patient 1: Fluoroscopic View Demonstrates Full Release of the Orthotopic Transcatheter Tricuspid Valve Replacement and Retraction of the Delivery System

Video 12 Patient 1: Multiplanar Reconstruction Imaging During Orthotopic Transcatheter Tricuspid Valve Replacement

Video 13 Patient 1: Tricuspid Leaflet Engagement During Orthotopic Transcatheter Tricuspid Valve Replacement

Video 14 Patient 1: Full Release of the Orthotopic Transcatheter Tricuspid Valve Replacement and Retraction of the Delivery System

Video 15 Patient 1: Three-Dimensional En Face View After Orthotopic Transcatheter Tricuspid Valve Replacement

Video 16 Patient 1: Transthoracic 4-Chamber Biplane View Revealing Mild to Moderate Prosthetic Valve Regurgitation

Video 17 Patient 2: Preprocedural Transesophageal Short-Axis Biplane View With Color Doppler

Video 18 Patient 2: Preprocedural Transesophageal Short-Axis Biplane Sweep

Video 19 Patient 2: Pull Test After Implantation of Anchor 13

Video 20 Patient 2: Pull Test After Implantation of Anchor 14

Video 21 Patient 2: Pull Test After Implantation of Anchor 15

Video 22 Patient 2: Three-Dimensional En Face View of the Tricuspid Valve Before Contraction of the Annuloplasty Device

Video 23 Patient 2: Three-Dimensional En Face View of the Tricuspid Valve After Cinching

Video 24 Patient 2: Transgastric Short-Axis Biplane Sweep After Implantation of the Annuloplasty Device

Video 25 Patient 2: Transesophageal Short-Axis Biplane View With Color Doppler After Transcatheter Annuloplasty Device Implantation

Video 26 Patient 2: Transesophageal Bicaval Biplane View After Caval Valve Implantation Demonstrating the Inferior Vena Cava Valve
